# Surgical treatment of anomalous origin of the left pulmonary artery from the descending aorta in a teenager: a case report

**DOI:** 10.3389/fcvm.2024.1423153

**Published:** 2024-09-09

**Authors:** Fuzheng Guo, Simeng Zhang, Zhe Du, Jing Tai, Fengbo Pei, Yi Shi

**Affiliations:** ^1^ Key Laboratory of Trauma and Neural Regeneration (Ministry of Education), Trauma Center, National Center for Trauma Medicine, Peking University People’s Hospital, Beijing, China; ^2^ Department of Cardiac Surgery, Peking University People’s Hospital, Beijing, China

**Keywords:** AOLPA, descending aorta, congenital heart disease, pulmonary hypertension, hemoptysis

## Abstract

Anomalous origin of one pulmonary artery (AOPA) is a rare congenital heart disease whose symptoms often occur in infancy, and patients have little chance of surviving into adulthood without timely treatments. AOPA is more frequent in infants and toddlers rather than in adults, and it accounts for only 0.12% of all congenital heart disease cases. In all AOPA cases, the right pulmonary artery from the ascending aorta remains common. This study reported a case with anomalous origin of the left pulmonary artery (AOLPA) from the descending aorta in a teenager who underwent double-incision surgery of median sternotomy and left lateral thoracotomies with favorable outcomes.

## 1 Introduction

As a kind of rare congenital heart disease (CHD), anomalous origin of one pulmonary artery (AOPA) was first proposed in 1868 by Fraentzel and it accounts for only 0.12% of all CHD cases. AOPA refers to one pulmonary artery branch originating from any part of the aorta and the other from the right ventricular outflow tract, and it more often involves the right pulmonary artery (70%-80%) than the left pulmonary artery (1, 2). AOPA is more common in infants, with 80% of the natural history occurring at less than 1 year; therefore, adult cases are extremely rare (3). Due to abnormal pulmonary blood flow, pediatric patients with AOPA often develop severe pulmonary hypertension (PH) in their early childhood, followed by right ventricular dysfunction and eventual death from refractory right heart failure (4). Early diagnosis and timely surgical treatment have been shown to significantly improve the prognosis of AOPA. The key point of surgical treatment is to separate the pulmonary artery of anomalous origin from the aorta and then reconnect it with the main pulmonary artery to restore normal anatomical relationship. According to previous adult case reports, anomalous origin of pulmonary artery is mainly located in the ascending aorta, and this can be treated with either median sternotomy or left lateral thoracotomies through a single-incision surgery. However, the patient in this case report had an anomalous origin of the left pulmonary artery (AOLPA) from the descending aorta, and results from computed tomography angiography (CTA) suggested that it would be difficult to operate on the descending aorta and the main pulmonary artery simultaneously under single incision. Therefore, we conducted double-incision surgery of median sternotomy and left lateral thoracotomies. The case details are discussed in the following section.

## 2 Case presentation

### 2.1 General information

**Figure 1:**
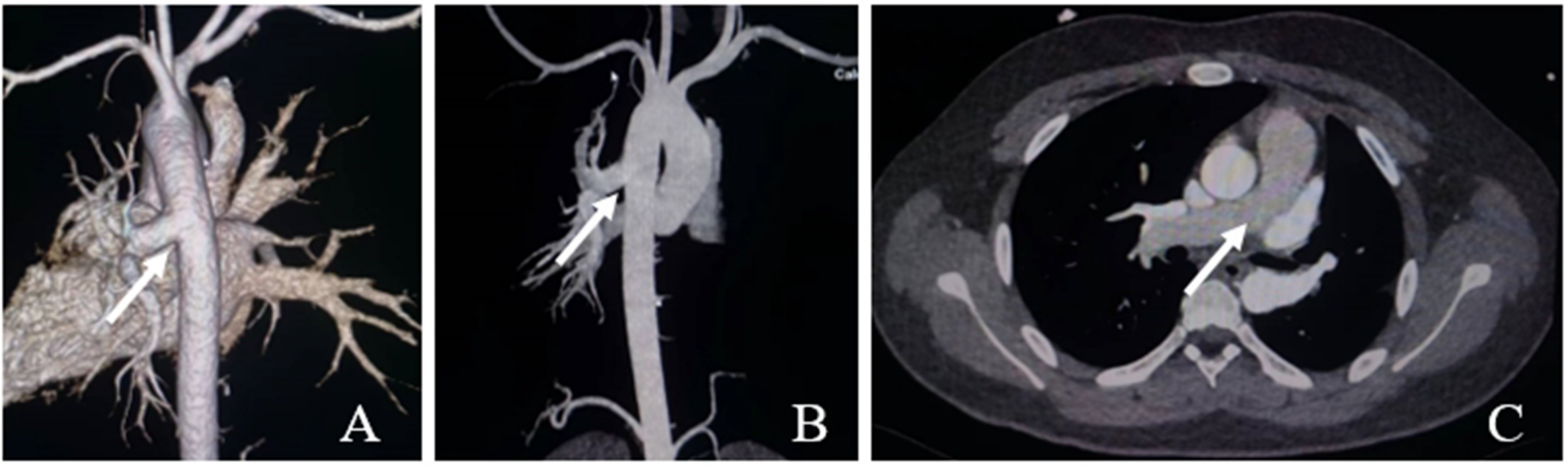
Ct aortic angiography. **(A,B)** The left pulmonary artery arises from the descending aorta (white arrow); **(C)** The main pulmonary artery continues directly into the right pulmonary artery (white arrow).

A 15-year-old male patient was admitted to our hospital in September 2022 because of hemoptysis following strenuous exercise around 4 years ago. The hemoptysis was bright red in color, about 5 ml each time, with aggravation for 2 months. He had no chest tightness, palpitations, cyanosis, or lower extremity edema. The patient showed no signs of systemic circulatory congestion and the right ventricular diameter was normal indicated by echocardiogram (Supplementary Material 1). Echocardiography revealed a branch of main pulmonary trunk to the right and moderate PH.; however, no left pulmonary artery branch was observed. Aortic CTA reported AOLPA from the thoracic aorta (Figure 1).

Physical examination: Well-developed with a height of 177 cm and weight of 90 kg. No cyanosis of the lips or deformity of the thorax was found. Heart rate was 72 beats/min, normal sinus rhythm No pathological murmur in any of the auscultatory valve areas, and resting finger pulse oxygen saturation was 98%. The right heart catheterization was performed and the mean pulmonary artery pressure was 15mmhg (Supplementary Material 2). Following the completion of various examinations, elective surgery was performed.

The preoperative discussion focused on two aspects. The first was the choice of surgical approach and method. The primary procedure for surgical treatment is to separate the pulmonary artery of anomalous origin from the aorta and then reconnect it with the main pulmonary trunk. Based on the results of the CTA and three-dimensional reconstruction technique, we found that the distance between the left pulmonary artery and the main pulmonary trunk after transection was too far to be directly anastomosed, and an artificial blood vessel was required to extend the pulmonary artery, followed by end-to-side anastomosis with the main pulmonary artery. However, due to the anatomical distance, it was difficult to deal with the descending aorta and the main pulmonary artery simultaneously under single incision. Therefore, we reached a consensus for a double-incision surgery of median sternotomy and left lateral thoracotomies. Nevertheless, attention should be paid to the pathophysiology associated with the pulmonary circulation. After the left pulmonary artery is reconnected to the main pulmonary artery, blood flow in the pulmonary circulation will be redistributed, potentially leading to hypoxemia. In addition, prolonged aortic blood supply to the left pulmonary artery may result in increased pulmonary artery resistance and slower pulmonary blood flow, increasing the risk of thrombosis in the pulmonary artery and artificial blood vessels. Therefore, we administered additional anticoagulant therapy for a certain period of time after surgery.

### 2.2 Surgical procedure

First, the patient was placed in the right lateral position. Following one-lung ventilation (OLV), a left fourth intercostal incision was made from the anterior axillary line to the angulus inferior scapulae line.

**Figure 2:**
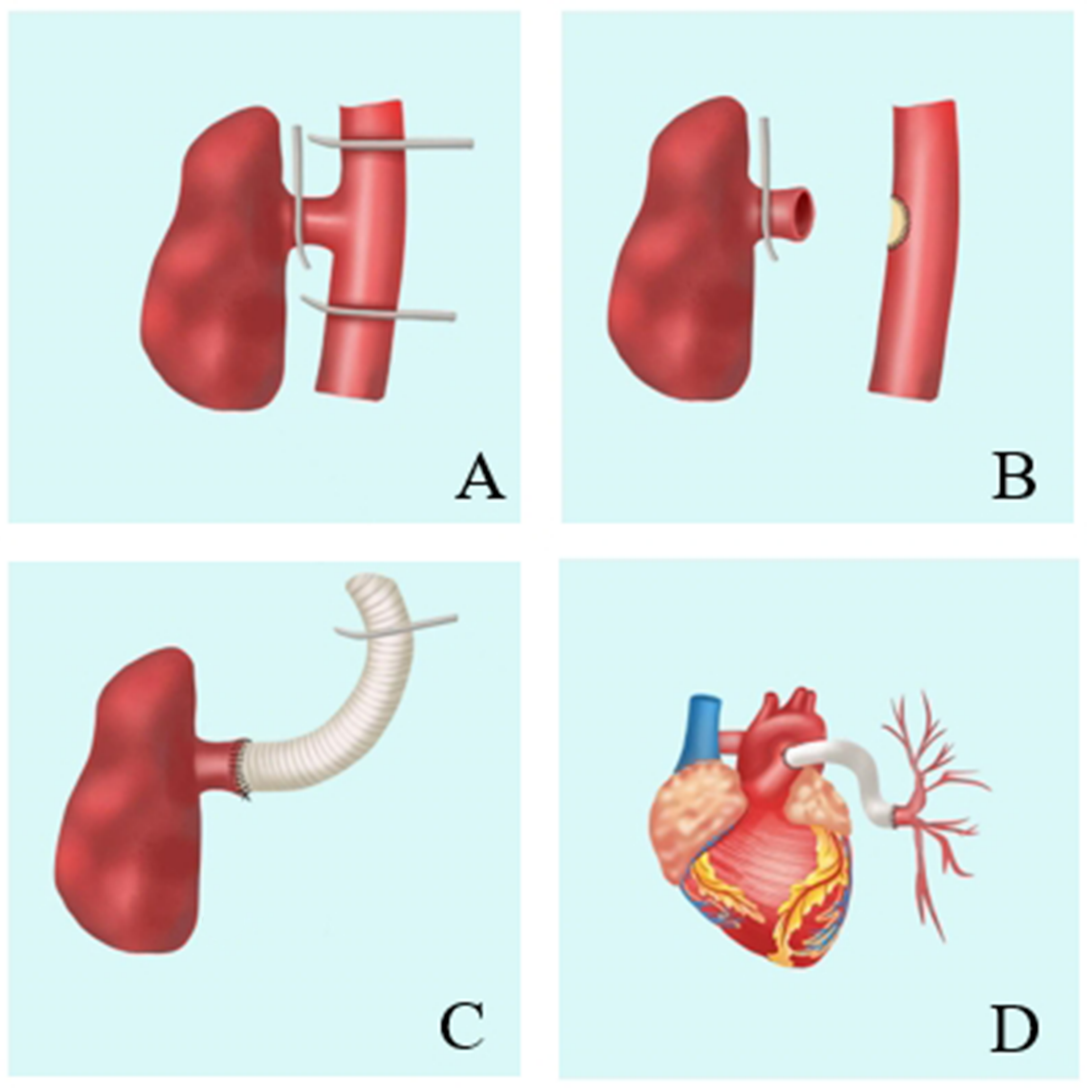
Repair of anomalous origin of left pulmonary artery from descending aorta. **A**: The left pulmonary artery and the descending aorta are blocked. **B**: Separating the left pulmonary artery and repairing of the descending aortic incision with a bovine pericardial patch. **C**: The anastomosis between artificial blood vessel and left pulmonary artery. **D**: The anastomosis between artificial blood vessel and pulmonary artery trunk.

Second, after entering the thoracic cavity, the descending aorta was fully exposed and the left pulmonary artery originating from the descending aorta was explored. The left pulmonary artery and descending aorta were fully freed, the intercostal artery adjacent to the surrounding was ligated, and the left pulmonary artery, as well as the proximal and distal descending aorta, were blocked (Figure 2A).

Third, the left pulmonary artery was separated and the descending aorta was opened following the repair of the descending aortic incision with a bovine pericardial patch (Figure 2B).

Fourth, 18# artificial blood vessel was used for end-to-end anastomosis with the left pulmonary artery. The right ventricular outflow tract was exposed after opening the pericardium. The distal end of the artificial blood vessel was ligated and then inserted into the pericardial cavity through the pericardial incision (Figure 2C).

Fifth, the left thoracic cavity was closed, and the patient was placed in the supine position.Once extracorporeal circulation was established following a median thoracotomy, the main pulmonary artery was incised and anastomosis of the artificial blood vessel and the main pulmonary artery was performed.

When tailoring the length of the artificial blood vessel, repeated alignment was performed before selecting the appropriate one to ensure the natural course of the left pulmonary artery in the state of lung recruitment and right ventricular filling. Meanwhile, both the upper and lower edges of the artificial blood vessel must be clear, corresponding to the anastomotic stoma of the left pulmonary artery to avoid distortion of the course.

Finally, the extracorporeal circulation machine was shut down smoothly, and the artificial blood vessel was well-shaped at the end of the operation (Figure 2D).

### 2.3 Follow-up

**Figure 3:**
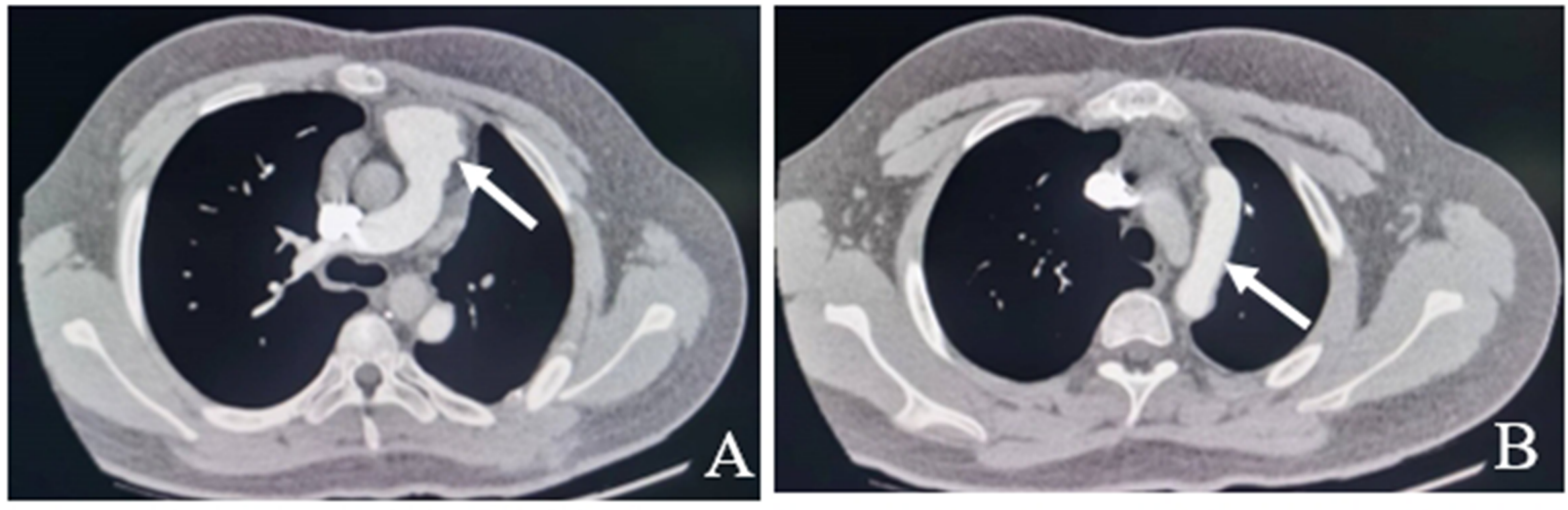
Pulmonary artery CT six months after the operation show the left pulmonary artery is well visualized without any obvious stenosis, dilatation, or filling defect (white arrow). **A**: No stenosis at the origin of the left pulmonary artery. **B**: No filling defects in artificial blood vessels.

Following the surgery, the patient was moved back to the intensive care unit (ICU). The patient had stable hemodynamics and was weaned from the ventilator 6 h after the surgery, with endotracheal tube being removed. The patient was then transferred from the ICU to the cardiac surgery ward where he received anticoagulation therapy with warfarin, which was continuously adjusted according to the international normalized ratio (INR). A repeat pulmonary artery CT six months after the operation showed that the left pulmonary artery was well visualized without any obvious stenosis, dilatation, or filling defect (Figure 3).

## 3 Discussion

AOPA is divided into three types based on its origins: type I from proximal to the ascending aorta, type II from the aortic arch or innominate artery, and type III from the descending aorta. AOPA often involves the right pulmonary artery originating from the ascending aorta, while the left pulmonary artery originating from the descending aorta is relatively rare. Currently, the etiology of AOPA is believed to be abnormal development of the sixth aortic arch during the early stage of embryonic development. The main pathophysiological change is that all the blood discharged by the right ventricle is injected into the unilateral lung, significantly increasing its blood flow, resulting in PH and, in severe cases, right heart failure (5). Based on the etiology and pathogenesis, the 2022 ESC/ERS Guidelines for the diagnosis and treatment of pulmonary hypertension proposed five clinical classifications of PH, among which group 5 (i.e., PH with unclear and/or multifactorial mechanisms) included PH caused by complex CHD (6). Currently, the mechanism of PH caused by AOPA remains unclear. Some studies have pointed out that blood in the aorta is directly perfused into pulmonary artery, significantly increasing pulmonary blood, leading to spasm of the pulmonary arterioles in the early stage and intimal thickening or medial fibrous hyperplasia in the later stage. Although both lungs are affected by different hemodynamics, similar pathophysiological changes can be observed in the pathological examination of the vessels on both sides, which may be related to the presence of communicating branches between the two lungs (7). The main clinical manifestation of this patient was hemoptysis following strenuous activity, the pathogenesis of which was associated with vasodilatation and destruction of the vascular media. Missed diagnosis and misdiagnosis of AOPA are common in clinical settings due to its low incidence. In patients with recurrent hemoptysis, combining right ventricular dysfunction and pulmonary hypertension of unknown cause, the possibility of AOPA should be considered.

Echocardiography and CTA for the aorta and pulmonary artery are important methods to confirm the diagnosis of AOPA, and they are also helpful in identifying the anatomical characteristics and assessing the severity. Once the diagnosis is confirmed, prompt surgical treatment is recommended in order to restore the normal anatomical relationship of the pulmonary artery. Only in the absence of Eisenmenger syndrome caused by PH may the surgery be performed. Since AOPA is more frequent in infants or toddlers rather than in adults, the condition should be fully evaluated to develop an appropriate treatment plan. The preoperative imaging in this case of AOLPA from the descending aorta suggested difficulty in dealing with the descending aorta and the main pulmonary artery simultaneously under single incision; therefore, we performed a double-incision surgery instead. Separation of the left pulmonary artery and descending aorta was performed through the left fourth intercostal incision, followed by an extension of the left pulmonary artery using an artificial blood vessel and anastomosis with the main pulmonary artery through a midline incision. Postoperative re-examination indicated that the left pulmonary artery blood flow was smooth, the main pulmonary artery was in a good position, and there was no evidence of pulmonary artery stenosis or thrombosis.

As a rare disease, AOPA is prone to missed diagnosis and misdiagnosis, particularly in cases with unexplained PH in children or adolescents. Clinical staff should take into consideration the possibility of AOPA. Echocardiography or right heart catheterization can help to better understand pulmonary arterial resistance. Following a comprehensive analysis of the surgical feasibility, surgical treatment should be performed as soon as possible. In terms of postoperative nursing, anticoagulation therapy in the early postoperative period and regular monitoring of pulmonary arterial blood flow are recommended.

## Data Availability

The raw data supporting the conclusions of this article will be made available by the authors, without undue reservation.
