# Supplementary material for: Surgical treatment of anomalous origin of the left pulmonary artery from the descending aorta in a teenager: a case report
Source: Front Cardiovasc Med. 2024 Sep 9;11:1423153. doi: 10.3389/fcvm.2024.1423153 (PMC11417616; doi:10.3389/fcvm.2024.1423153)
Supplement: Supplementary file 1 [file Datasheet1.pdf]

Supplementary 1   The preoperative echo

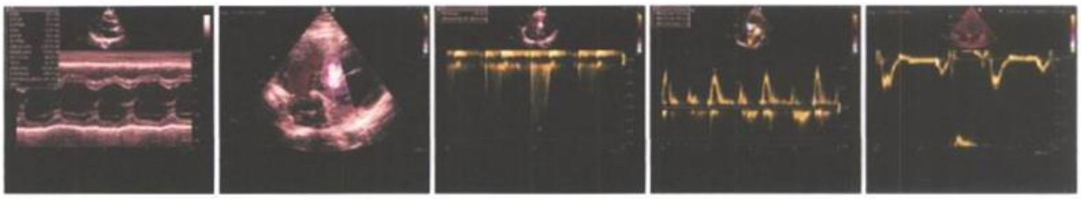

M型/二维测量和计算

主肺动脉内径: 2.2 厘米
主动脉根部内径: 3.1 厘米
升主动脉内径: 2.6 厘米
左房前后径: 3.7 厘米

室间隔舒张末期厚度: 0.90 厘米
左室舒张末期内径: 6.2 厘米
左室收缩末期内径: 4.2 厘米
左室后壁舒张末期厚度: 1.0 厘米
LV mass(C)d: 250.3 克

射血分数(Teich): 58.8 %
舒张末期容积(Teich): 193.8 毫升
收缩末期容积(Teich): 79.8 毫升

多普勒测量和计算

主动脉瓣上最大流速: 154.0 厘米/秒
主动脉峰值压力阶差: 9.5 mmHg

二尖瓣E峰速度: 96.6 厘米/秒
二尖瓣A峰速度: 63.7 厘米/秒
二尖瓣E/A值: 1.5
二尖瓣环室间隔 E' 峰值速率: 8.8 厘米/秒
二尖瓣室间隔E/E': 10.9

三尖瓣反流最大流速: 288.9 厘米/秒
三尖瓣反流最大压差: 33.4 mmHg
肺动脉最大流速: 119.6 厘米/秒
肺动脉峰值压力阶差: 5.7 mmHg

大血管 主动脉根部内径正常。 肺动脉主干内径正常。 可见肺动脉主干向右发出一个分支，未见明确左肺动脉分支。
心房 左房内径正常。 右房内径正常。 房间隔连续性完整。
左心室 左室扩大。 左室壁厚度正常。 静息状态下左室壁收缩活动未见明显异常。 室间隔连续性完整。
右心室 右室内径正常。
二尖瓣 二尖瓣结构和功能未见明显异常。 CDFI: 二尖瓣未见反流。
三尖瓣 三尖瓣结构和功能未见明显异常。 CDFI: 三尖瓣轻微反流。 连续多普勒估测肺动脉收缩压为38mmHg。
主动脉瓣 主动脉瓣结构和功能未见明显异常。 CDFI: 主动脉瓣未见反流。
肺动脉瓣 肺动脉瓣结构和功能未见明显异常。
心包 心包未见增厚。 心包腔内未见无回声区或其他异常回声。

结论

左肺动脉缺如? 起源异常?
左室扩大

PAD	2.2cm	LVDD	6.2cm
AOD	3.1cm	LVEF	58.8%
SPAP	38mmhg	LAD	3.7cm

Supplementary 2 The right heart catheterization

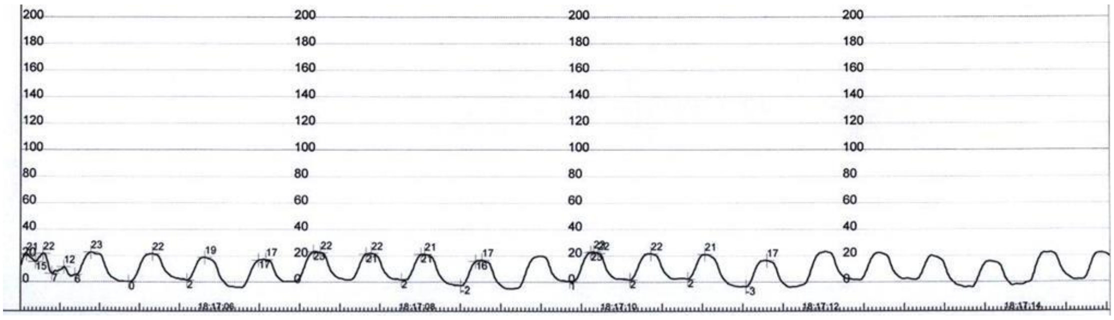

Right ventricular pressure

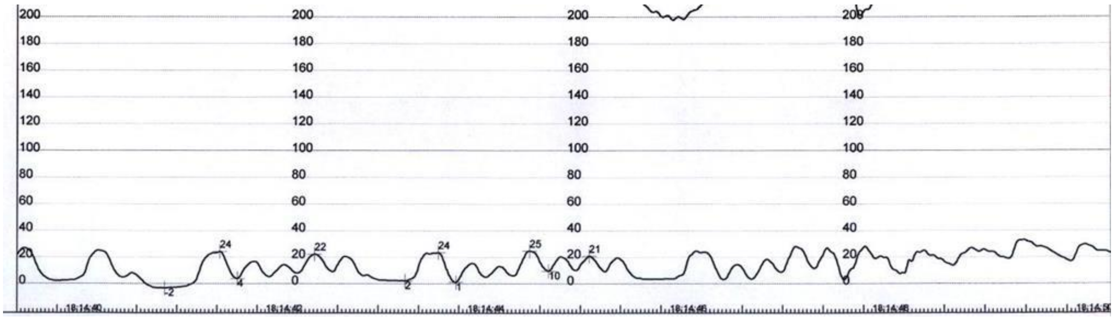

Main pulmonary artery pressure

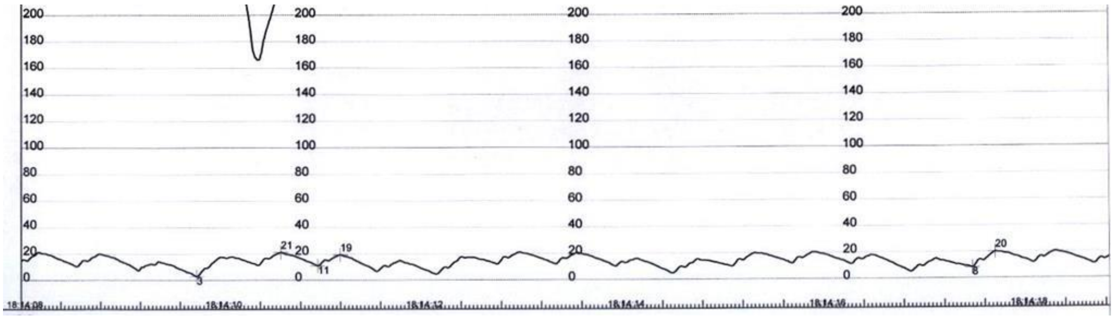

Right pulmonary artery pressure

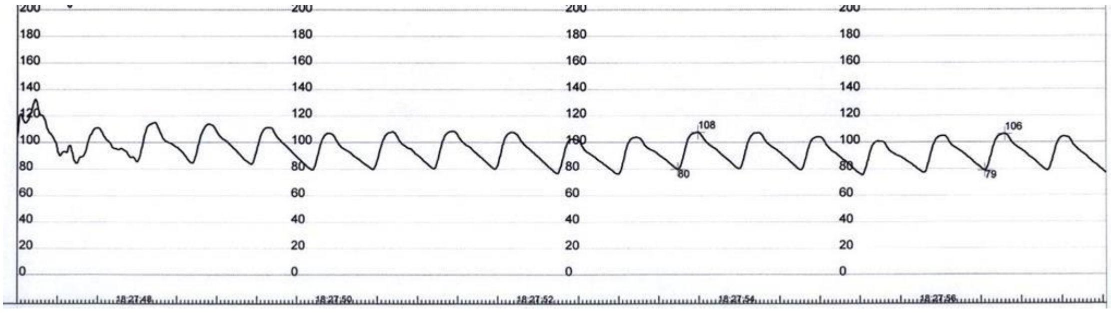

Left pulmonary artery pressure

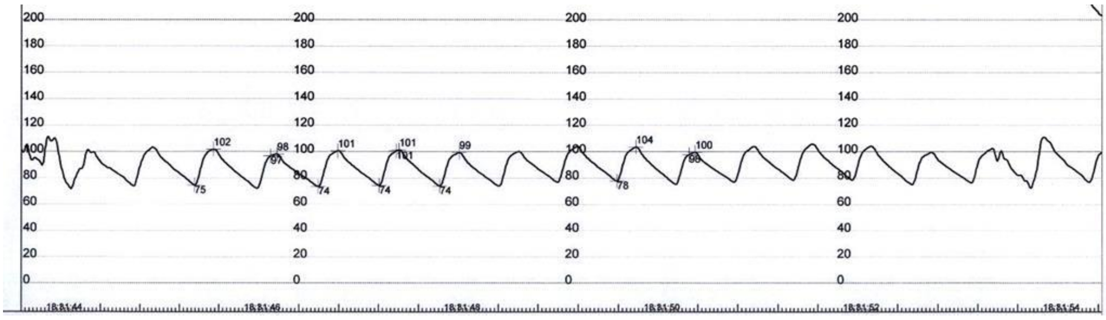

Descending aorta pressure

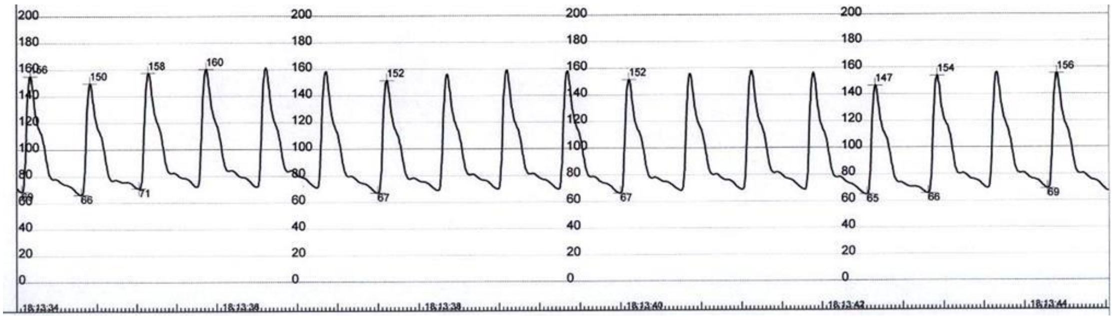

Femoral artery pressure

Supplementary 3

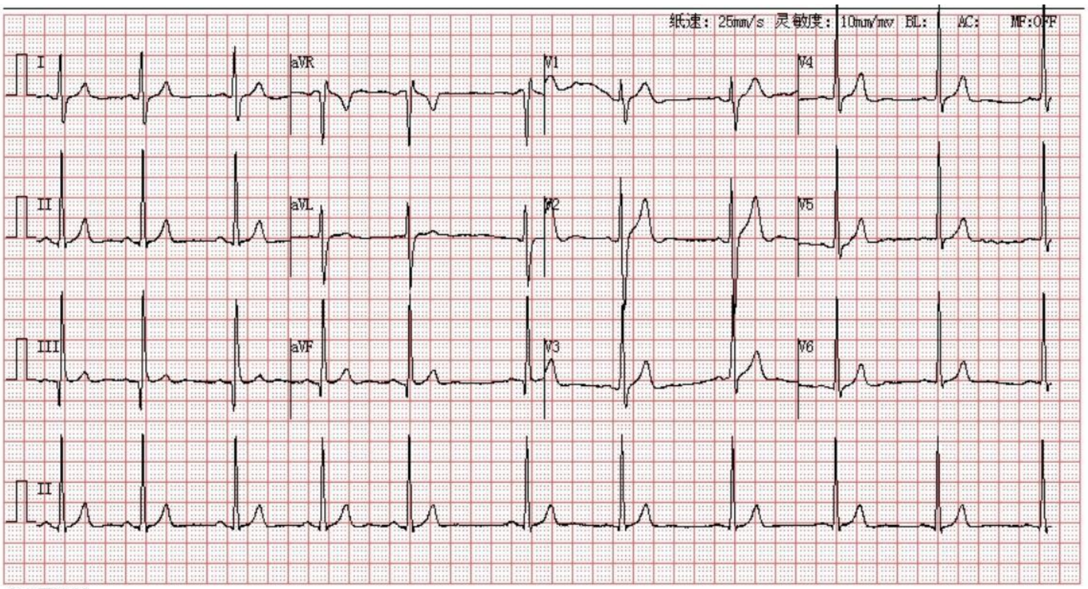

心电图诊断：

窦性心律不齐

Supplementary 4   chest X-ray

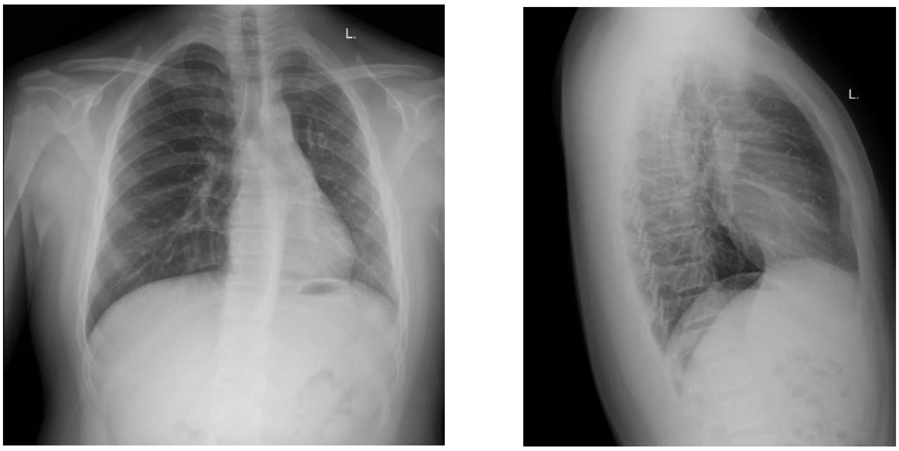

Supplementary 5

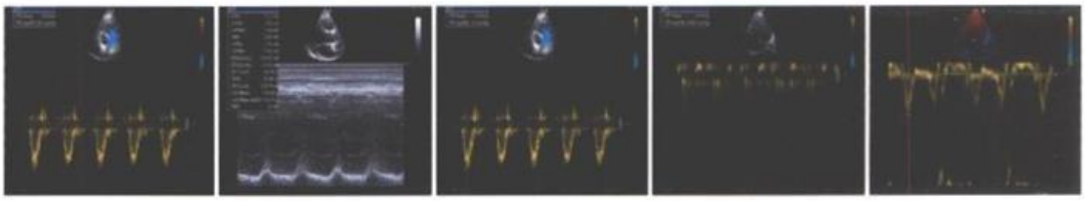

M型/二维测量和计算

主肺动脉内径: 2.8 厘米
主动脉根部内径: 2.7 厘米
升主动脉内径: 2.8 厘米
左房前后径: 3.5 厘米

室间隔舒张末期厚度: 0.73 厘米
左室舒张末期内径: 5.6 厘米
左室收缩末期内径: 3.6 厘米
左室后壁舒张末期厚度: 0.79 厘米
LV mass(C)d: 154.3 克

射血分数(Teich): 64.8 %
舒张末期容积(Teich): 152.6 毫升
收缩末期容积(Teich): 53.8 毫升

多普勒测量和计算

主动脉瓣上最大流速: 135.3 厘米/秒
主动脉峰值压力阶差: 7.3 mmHg

二尖瓣E峰速度: 66.5 厘米/秒
二尖瓣A峰速度: 50.9 厘米/秒
二尖瓣E/A值: 1.3
二尖瓣环室间隔 E' 峰值速率: 9.8 厘米/秒
二尖瓣室间隔E/E': 6.8

三尖瓣反流最大流速: 244.9 厘米/秒
三尖瓣反流最大压差: 24.0 mmHg
肺动脉最大流速: 86.2 厘米/秒
肺动脉峰值压力阶差: 3.0 mmHg

大血管 主动脉根部内径正常。 肺动脉主干增宽。
心房 左房内径正常。 右房内径正常。 房间隔连续性完整。
左心室 左室内径正常。 左室壁厚度正常。 静息状态下左室壁收缩活动未见明显异常。 室间隔连续性完整。
右心室 右室内径正常。
二尖瓣 二尖瓣结构和功能未见明显异常。 CDFI: 二尖瓣未见反流。
三尖瓣 三尖瓣结构和功能未见明显异常。 CDFI: 三尖瓣轻微反流。 连续多普勒估测肺动脉收缩压为29mmHg。
主动脉瓣 主动脉瓣结构和功能未见明显异常。 CDFI: 主动脉瓣未见反流。
肺动脉瓣 肺动脉瓣结构和功能未见明显异常。
心包 心包未见增厚。 心包腔内未见无回声区或其他异常回声。

结论

肺动脉内径增宽

PAD	2.8cm	LVDD	5.6cm
AOD	2.8cm	LVEF	64.8%
SPAP	29mmhg	LAD	3.5cm
